# Prognostic Value Of Deep Learning Based RCA PCAT and Plaque Volume Beyond CT-FFR In Patients With Stent Implantation

**DOI:** 10.2174/0115734056335065250426150739

**Published:** 2025-05-12

**Authors:** Zengfa Huang, Ruiyao Tang, Xinyu Du, Yi Ding, ZhiWen Yang, Beibei Cao, Mei Li, Xi Wang, Wanpeng Wang, Zuoqin Li, Jianwei Xiao, Xiang Wang

**Affiliations:** 1 Department of Radiology, The Central Hospital of Wuhan, Tongji Medical College, Huazhong University of Science and Technology, Wuhan, Hubei, 430014, China; 2 Department of Radiology, The Central Hospital of Wuhan Base, Hubei University of Medicine, Shiyan, Hubei, 442000, China; 3 ShuKun Technology Co., Ltd., Beijing, 100029, China; 4 Department of Community Health, Hanyang District Center For Disease Control and Prevention, Wuhan, Hubei, 430050, China

**Keywords:** Coronary computed tomography angiography, Pericoronary adipose tissue attenuation computed tomography, Percutaneous coronary intervention, Prognosis, Artificial intelligence, Coronary artery disease, Myocardial infraction

## Abstract

**Aim:**

The study aims to investigate the prognostic value of deep learning based pericoronary adipose tissue attenuation computed tomography (PCAT) and plaque volume beyond coronary computed tomography angiography (CTA) -derived fractional flow reserve (CT-FFR) in patients with percutaneous coronary intervention (PCI).

**Methods:**

A total of 183 patients with PCI who underwent coronary CTA were included in this retrospective study. Imaging assessment included PCAT, plaque volume, and CT-FFR, which were performed using an artificial intelligence (AI) assisted workstation. Kaplan-Meier survival curves analysis and multivariate Cox regression were used to estimate major adverse cardiovascular events (MACE), including non-fatal myocardial infraction (MI), stroke, and mortality.

**Results:**

In total, 22 (12%) MACE occurred during a median follow-up period of 38.0 months (34.6-54.6 months). Kaplan-Meier analysis revealed that right coronary artery (RCA) PCAT (*p* = 0.007) and plaque volume (*p* = 0.008) were significantly associated with the increase in MACE. Multivariable Cox regression indicated that RCA PCAT (hazard ratios (HR): 7.05, 95%CI: 1.44-34.63, p = 0.016) and plaque volume (HR: 3.84, 95%CI: 1.44-10.27, p = 0.007) were independent predictors of MACE after adjustment by clinical risk factors. However, CT-FFR was not independently associated with MACE in multivariable Cox regression (p = 0.150).

**Conclusions:**

Deep learning based RCA PCAT and plaque volume derived from coronary CTA were found to be more strongly associated with MACE than CT-FFR in patients with PCI.

## 1 INTRODUCTION

Percutaneous coronary intervention (PCI) is recommended as the first-line treatment for patients with high-complexity coronary artery disease (CAD) in ESC and AHA guidelines [1, 2]. More than 500,000 PCI procedures are performed annually worldwide for stable CAD [3]. According to the statistical data from the National Center for Cardiovascular Disease (NCCD), the total number of registered cases of PCI therapy in mainland China was 1,164,117, which is an increase of 20.18% from 2020 [4]. However, the incidence of MACE, including myocardial infarction, revascularizations, and all-cause mortality after PCI, remains up to 5% to 15% [5]. In addition, a previous study has reported that the recurrence rate of chest pain is up to 50% [6]. Previous studies have used clinical models and the SYNTAX II score to evaluate the prognosis in patients with PCI [7, 8]. Unfortunately, so far, limited studies have reported an effective method that contains coronary computed tomography angiography (CTA) features to assess the prognostic value in those patients [9].

Coronary CTA was regarded as the first-line examination for evaluation of patients with PCI. Recently, coronary CTA -derived fractional flow reserve (CT-FFR), plaque volume, and perivascular fat attenuation index have been introduced as a novel imaging biomarker in patients with CAD. Previous studies have indicated that post-PCI invasive FFR values revealed a close association with repeat PCI and poor prognosis during follow-up [10]. Furthermore, a recent study with a small sample has shown that CT-FFR was highly correlated with FFR and showed good prognostic value in predicting MACE in patients with PCI [11]. Another novel imaging biomarker measured in coronary CTA is the perivascular fat attenuation index (FAI), which reflects the coronary vascular inflammation. The increased FAI value was reported to be closely related to the high risk of MACE [12, 13]. Recent coronary CTA studies have demonstrated that plaque volume provides independent further MACE events prediction during follow-up [14, 15]. However, few studies focus on the difference between CT-FFR, FAI, and plaque volume for predicting MACE in patients with PCI. Thus, the present study aimed to investigate the prognostic value of deep learning based CT-FFR, pericoronary adipose tissue attenuation computed tomography (PCAT), and plaque volume in patients with PCI.

## 2 MATERIALS AND METHODS

### 2.1 Study Patient

The retrospective and observational study complied with the Declaration of Helsinki. The study protocol was approved by the institutional ethics committee, and written informed consent was waived by the institutional ethics committee because of its retrospective observational nature of the study. Between November 2018 and December 2020, consecutive patients who underwent coronary CTA for evaluating percutaneous coronary intervention (PCI) were retrospectively enrolled from our two hospitals (Nanjing Road and Houhu districts). The inclusion criterion was age above 18 years. Exclusion criteria were history of myocardial infraction, coronary revascularization (coronary artery bypass grafting, CABG), low-quality coronary CTA images, missing coronary CTA images or reports, or reports without stenosis information, missing CT-FFR values, or loss of follow-up.

### 2.2 Coronary CTA Acquisition Protocol

Coronary CTA examinations were performed with prospectively or retrospectively ECG-triggered on a dual-scanner CT scanner (Somatom Definition, Siemens Medical Solutions, Forchheim, Germany) or ICT scanner (Philips Brilliance 64, Philips Medical Systems, Best, the Netherlands). Detailed coronary CTA protocol and parameters were presented in previous reports [16, 17].

### 2.3 CT-FFR, PCAT CT Attenuation, Plaque Volume Measurement and Analysis

CT-FFR values were calculated through commercial software based on a deep learning algorithm, which has been described in our previous reports [16]. Plaque volume and PCAT measurements were performed on an AI ML platform (CoronaryDoc Premium and Perivascular Fat Analysis Tool,Shukun Technology Co, Beijing, China), and the total plaque volume was the sum of the separate plaque volumes in each coronary artery segment.

### 2.4 Follow up

The local institutional review boards approved the follow-up procedures of the study. The primary endpoint was defined as MACE, including MI, stroke, and mortality. One of our team queried the local Community Health Service Centers to record the MACE status. MACE was confirmed through medical records or by contacting the patient’s family members once it occurred outside of the city. Two experts in the centers for disease control and prevention (CDC) performed the process of the follow-up with blinding to basic, clinical, and coronary CTA information of patients. The deadline date of the follow-up was September 30, 2022.

### 2.5 Statistical Analysis

Continuous and categorical variables were presented as mean (± SD) and frequencies (percentages), respectively. Student’s t-test and chi-square test were used for comparisons between groups in continuous variables and categorical variables, respectively. The Kaplan-Meier method with log-rank test was presented to calculate the Cumulative event-free survival between groups. Then, univariate and multivariate Cox regression were performed to calculate the Hazard ratio (HR) with 95% confidence intervals (95% CI). Multivariate Cox analysis was adjusted with basic and clinical information, including age, gender, alcohol consumption, smoking status, hypertension and diabetes. The maximally selected rank statistics method was used to evaluate the prognostic threshold of PCAT and plaque volume. *P* < 0.05 was considered to be statistically significant. All statistical analyses were performed using R statistical package (version 4.3, R foundation for Statistical Computing, Vienna, Austria), SPSS (version 18, SPSS, Inc., Chicago, IL, USA), and MedCalc Statistical Software (version16.8.4 Ostend, Belgium).

## 3 RESULTS

### 3.1 Patient Basic Characteristics and Coronary CTA Features

A total of 183 patients with PCI who underwent coronary CTA were included in the final analyses, and 22 patients occurred MACE during a median follow-up period of 38.0 months (IQR 34.6-54.6 months). Overall, the average age of 183 patients was 68.1 ± 10.3 years, with 111 (60.7%) male patients. Patients occurred MACE showed significantly higher percentage rate in plaque volume > 20 mm^3^ (14 (63.6%) vs. 56 (34.8%), *p* = 0.009) and PCAT_RCA > -64 HU (4 (18.2%) vs. 7 (4.3%), *p* = 0.010) than those in patients without MACE. However, CT-FFR, PCAT _LAD, PCAT _LCX, and PCAT volume showed no significant differences between patients with and without MACE. The patients’ characteristics and coronary CTA features were displayed in Table **1**.

### 3.2 CT-FFR, PCAT CT Attenuation and Plaque Volume with MACE

**Fig. (1A-B):**
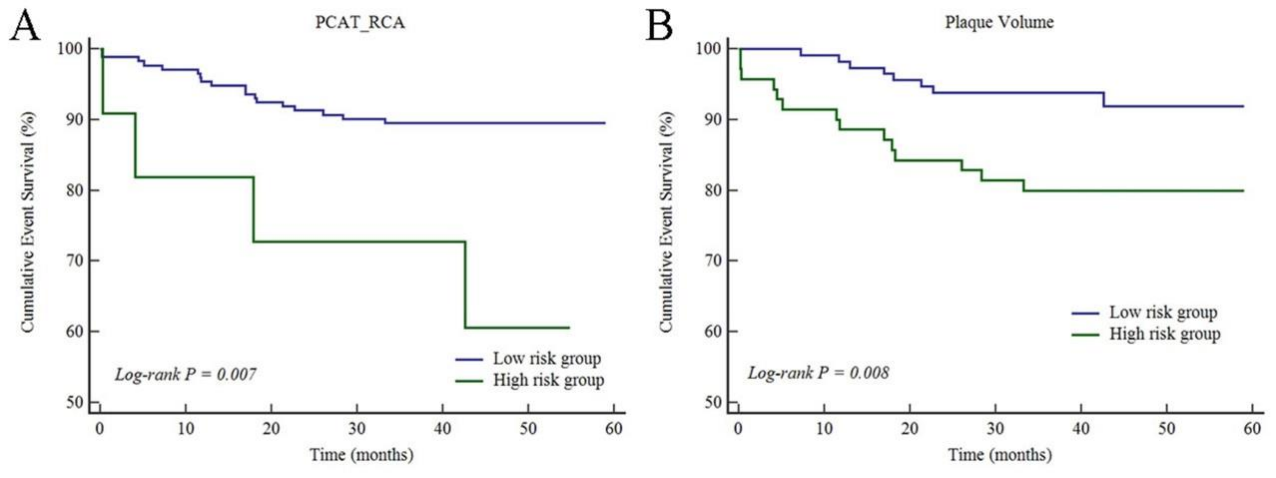
Cumulative event survivals of PCAT_RCA and plaque volume in the study patients.

**Fig. (2A-B):**
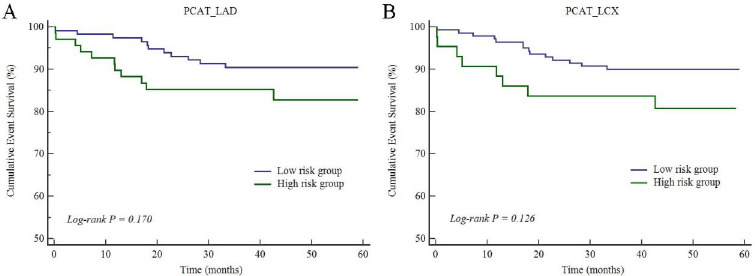
Cumulative event survivals of PCAT_LAD and PCAT_LCX in the study patients.

**Fig. (3):**
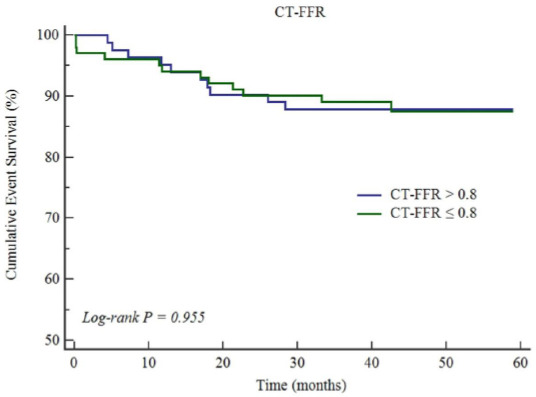
Cumulative event survivals of CT-FFR in the study patients.

The prognostic threshold of PCAT CT attenuation was > -77 HU for LAD, > -67 HU for LCX, > -64 HU for RCA, respectively, according to Youden index analysis. Youden index analysis showed that plaque volume > 20 mm^3^ was the prognostic threshold of MACE. The Kaplan-Meier survival curves indicated that plaque volume and PCAT_RCA CT attenuation showed a close association with the increasing rate of MACE (*p* = 0.008 for plaque volume and *p* = 0.007 for PCAT_RCA, Fig. **1A,B**). However, PCAT_LAD and PCAT_LCX CT attenuation (Fig. **2A,B**), as well as CT-FFR, showed no association with the increasing rate of MACE (Fig. **3**).

### 3.3 Univariable and Multivariable Cox Regression Analysis

**Fig. (4):**
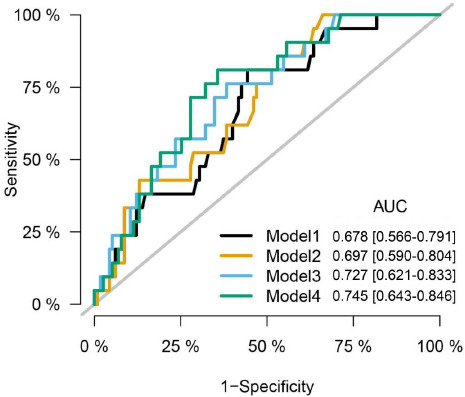
The area under ROC curve for predicting MACE in four models. Model 1 = clinical model (age, gender, smoke, drink, hypertension, diabetes); Model 2 = clinical model + PCAT_RCA CT attenuation; Model 3 = clinical model + plaque volume; Model 4 = clinical model + PCAT_RCA + plaque volume.

Univariable Cox regression analysis showed that PCAT_RCA CT attenuation (*p* = 0.013) and plaque volume (*p* = 0.012) were associated with MACE (Table **2**). In multivariable Cox regression analysis, after adjusting clinical risk factors (age, gender, smoke, drink, hypertension, diabetes), PCAT_RCA CT attenuation was associated with MACE in model 1 (HR, 4.03; 95% CI: 1.16-13.98, *p* = 0.028) and plaque volume was associated with MACE in model 2 (HR, 2.63; 95% CI: 1.08-6.44, *p* = 0.034). After adjusting clinical risk factors and all coronary CTA features, PCAT_RCA CT attenuation (HR, 7.05; 95% CI: 1.44-34.63, *p* = 0.016) and plaque volume (HR, 3.84; 95% CI: 1.44-10.27, *p* = 0.007) remained independent significant predictors of MACE in model 3 (Table **3**). The area under ROC curve (AUC) for predicting MACE was 0.678 (95% CI: 0.566-0.791), 0.697 (95% CI: 0.590-0.804), 0.727 (95% CI: 0.621-0.833), 0.745 (95% CI: 0.643-0.846) for clinical model (age, gender, smoke, drink, hypertension, diabetes), clinical model + PCAT_RCA CT attenuation, clinical model + plaque volume and clinical model + PCAT_RCA CT attenuation + plaque volume, respectively (Fig. **4**).

## 4 DISCUSSION

As far as we know, this is the first study to explore the association between deep learning based CT-FFR, PCAT CT attenuation, plaque volume, and MACE in patients with PCI. The current study investigated the prognostic potential of deep learning based CT-FFR, PCAT CT attenuation, and plaque volume in patients with PCI. Our results demonstrated that PCAT_RCA CT attenuation and plaque volume were independently associated with the increasing risk of MACE.

Previous studies have elaborated the prognostic value of invasive FFR (iFFR). The FAME (Fractional Flow Reserve Versus Angiography for Multivessel Evaluation) 1 and 2 studies revealed that a higher post-PCI iFFR value predicted a better clinical outcome [18]. Furthermore, Angarwal *et al* indicated that post-PCI iFFR showed incremental prognostic value beyond clinical and angiographic factors in predicting MACE [19]. In addition, meta-analysis has demonstrated that post-PCI iFFR value revealed an inverse relationship with composite MACE (MI, death, revascularization) [20]. However, the prognostic value of deep learning based CT-FFR in patients with PCI has not been clearly explored. The present study showed that deep learning based CT-FFR was not associated with MACE in patients with PCI.

PCAT CT attenuation has been regarded as an imaging biomarker for capturing coronary inflammation in coronary CTA. Previous studies have investigated the prognostic value of PCAT CT attenuation [12, 13, 21, 22].the Cardiovascular RISk Prediction using Computed Tomography (CRISP-CT) study showed that PCAT_RCA CT attenuation (HR, 1.49-1.84), PCAT_LAD CT attenuation (HR, 1.77-1.78) and PCAT_LCX CT attenuation (HR, 1.37-1.47) were independently associated with all-cause death in both derivation and validation cohorts during a median follow-up of 72 months (derivation cohort) and 54 months (validation cohort), respectively [12]. However, recent studies revealed that only PCAT_RCA CT attenuation present associated with poor clinical outcome. Diemen *et al* revealed that PCAT_RCA CT attenuation remained as an independent predictor after adjusting for clinical and imaging factors (HR: 2.45, 95%CI: 1.23-4.93, p=0.011), whereas PCAT_LAD and PCAT_LCX CT attenuation were not associated with the endpoint [21]. Tzolos *et al* further indicated that PCAT_RCA CT attenuation, not PCAT_LAD or PCAT_LCX CT attenuation, was predictive of further MI [22]. The present study also indicated that only PCAT_RCA CT attenuation was independently correlated with MACE when adjustment of clinical and imaging factors was performed in patients with PCI. The possible reason for this phenomenon may be because there is more fat structure around RCA compared to LAD and LCX. In addition, compared with LAD and LCX, there are fewer side branches of RCA. These together made the measurement and analysis of PCAT CT attenuation easier [13, 22].

Plaque volumes derived from coronary CTA have been demonstrated to have high prognostic value for adverse cardiovascular events [23-25]. Some studies revealed that this prognostic value was higher than clinical risk and lumen stenosis factors [26-28]. The present study showed similar results. The current measurements of plaque volume in coronary CTA were mainly dependent on various semi-automated research software. Although these platforms showed high correlations with intravascular ultrasound, the measurements and analysis of plaque volume are time-consuming, as this requires a large amount of manual input from expert readers [25]. Therefore, it limited its implementation in clinical practice. Our deep learning based-plaque volume measurement improves the process time-saving, thus increasing its potential for clinical application in the future. The present results indicated that plaque volumes of the coronary tree quantified by automatic measurement have an independent and strong prognostic value for MACE in patients with PCI, which has not been reported previously. Moreover, we determined an optimum cutoff (> 20 mm^3^), exceeding this value leads to a sharp increase in the risk of events.

There are some limitations in the current study. First, the current study is a single-center retrospective study. This study lacks information on lifestyle changes, such as exercise, sleep, or dietary habits, and medical therapy after coronary CTA examination, this information may influence future outcomes in the present study, thus may lead to biased results. Second, the high-risk plaque (HRP) has not been evaluated because the current AI version still has shortcomings in identifying and interpreting HRP. Third, other widely used classifications based on cardio CT scan (coronary artery calcification scores, CACS), or clinical information (SYNTAX score) have not been included. Further large multi-center investigations are needed to determine our findings.

## CONCLUSION

In conclusion, deep learning based RCA PCAT and plaque volume derived from coronary CTA, not CT-FFR, was found to be associated with MACE in patients with PCI.

## Figures and Tables

**Table 1:** Baseline characteristic and Coronary CTA features stratified by the occurrence of MACE.

**Variables**	**Total (N=183)**	**Without MACE (N=161)**	**With MACE (N=22)**	**P-value**
Age (years)	68.1 ± 10.3	67.6 ± 10.1	71.2 ± 10.9	0.128
Gender (Male, n, %)	111 (60.7)	97 (60.2)	14 (63.6)	0.760
Smoke	51 (27.9)	44 (27.3)	7 (31.8)	0.660
Drink	27 (14.8)	24 (14.9)	3 (13.6)	0.875
Hypertension	138 (75.4)	118 (73.3)	20 (90.9)	0.072
Diabetes	78 (42.6)	66 (41.0)	12 (54.5)	0.228
Ejection Fraction (%)	58.4 (4.9)	58.6 (4.5)	57.2 (7.3)	0.220
Medication				
Anti-hypertensive	153 (83.6)	133 (82.6)	20 (90.9)	0.324
Statins	172 (94.0)	151 (93.8)	21 (95.5)	0.758
Anti- coagulants	177 (96.7)	155 (96.3)	22 (100.0)	0.357
Anti-angina	53 (29.0)	44 (27.3)	9 (40.9)	0.188
Anti-diabetes	67 (36.6)	57 (35.4)	10 (45.5)	0.359
Anti-heart failure	12 (6.6)	10 (6.2)	2 (9.1)	0.609
Coronary CTA features				
PCI vessel number	1.2 (0.5)	1.2 (0.5)	1.2 (0.4)	0.943
PCI target vessel				0.244
LAD	134	117	17	
LCX	33	26	7	
RCA	38	35	3	
CT-FFR ≤0.80	101 (55.2)	89 (55.3)	12 (54.5)	0.948
Plaque Volume >20 mm^3^	70 (38.3)	56 (34.8)	14 (63.6)	0.009
FAI_LAD >-77 HU	68 (37.2)	57 (35.4)	11 (50.0)	0.184
FAI_LCX >-67 HU	43 (23.5)	35 (21.7)	8 (36.4)	0.129
FAI_RCA >-64 HU	11 (6.0)	7 (4.3)	4 (18.2)	0.010
Volume_ PCAT_LAD >1006 mm^3^	172 (94.0)	150 (93.2)	22 (100)	0.206
Volume_ PCAT_LCX >1754 mm^3^	34 (18.6)	29 (18.0)	5 (22.7)	0.594
Volume_ PCAT_RCA >1663 mm^3^	159 (86.9)	140 (87.0)	19 (86.4)	0.938

**Abbreviations:** PCAT = pericoronary adipose tissue attenuation; CT-FFR = coronary computed-derived fractional flow reserve; LAD = left anterior descending; LCX = left circumflex branch; RCA = right coronary artery; HR = Hazard ratio; CI = confidence intervals; HU = Hounsfield unit; MACE = major adverse cardiovascular events.

**Table 2:** Univariable Cox regression analysis for predicting MACE.

**Variables**	**Univariate**	
	**HR (95% CI)**	**P-value**
Age	1.03 (0.99-1.08)	0.120
Gender (male)	1.15 (0.48-2.75)	0.750
Smoke	1.24 (0.50-3.03)	0.642
Drink	0.90 (0.27-3.03)	0.861
Hypertension	3.41 (0.90-14.60)	0.098
Diabetes	1.64 (0.71-3.79)	0.250
CT-FFR ≤0.80	0.96 (0.41-2.21)	0.916
Plaque Volume >20 mm^3^	3.06 (1.28-7.29)	0.012
PCAT_LAD >-77 HU	1.78 (0.77-4.11)	0.176
PCAT_LCX >-67 HU	1.95 (0.82-4.65)	0.134
PCAT_RCA >-64 HU	3.98 (1.35-11.78)	0.013
Volume_ PCAT_LAD >1006 mm^3^	22.25 (0.01-40781)	0.418
Volume_ PCAT_LCX >1754 mm^3^	1.36 (0.50-3.69)	0.544
Volume_ PCAT_RCA >1663 mm^3^	1.01 (0.30-3.43)	0.982

**Abbreviations:** PCAT = pericoronary adipose tissue attenuation; CT-FFR = coronary computed-derived fractional flow reserve; LAD = left anterior descending; LCX = left circumflex branch; RCA = right coronary artery; HR = Hazard ratio; CI = confidence intervals; HU = Hounsfield unit; MACE = major adverse cardiovascular events.

**Table 3:** Multivariate Cox regression analysis for predicting MACE.

**Variables**	**Model 1**		**Model 2**		**Model 3**	
	**HR (95% CI)**	**P-value**	**HR (95% CI)**	**P-value**	**HR (95% CI)**	**P-value**
Age	1.04 (0.99-1.09)	0.683	1.03 (0.98-1.08)	0.274	1.01 (0.96-1.07)	0.683
Gender (male)	1.04 (0.40-2.70)	0.571	1.10 (0.43-2.84)	0.846	0.73 (0.25-2.16)	0.571
Smoke	1.05 (0.32-3.45)	0.935	1.64 (0.54-4.95)	0.382	1.49 (0.45-4.95)	0.519
Drink	1.24 (0.27-5.70)	0.785	0.67 (0.15-3.09)	0.606	1.00 (0.18-5.51)	0.999
Hypertension	3.08 (0.71-13.40)	0.133	3.00 (0.69-13.02)	0.143	3.86 (0.74-20.27)	0.110
Diabetes	1.55 (0.66-3.66)	0.317	1.38 (0.59-3.23)	0.460	1.42 (0.58-3.48)	0.445
CT-FFR ≤0.80					0.48 (0.18-1.31)	0.150
Plaque Volume >20 mm^3^			2.63 (1.08-6.44)	0.034	3.84 (1.44-10.27)	0.007
PCAT_LAD >-77 HU					1.50 (0.48-4.67)	0.484
PCAT_LCX >-67 HU					1.37 (0.35-5.37)	0.650
PCAT_RCA >-64 HU	4.03 (1.16-13.98)	0.028			7.05 (1.44-34.63)	0.016
Volume_ PCAT_LAD >1006 mm^3^					-	0.973
Volume_ PCAT_LCX >1754 mm^3^					1.90 (0.63-5.76)	0.256
Volume_ PCAT_RCA >1663 mm^3^					0.90 (0.22-3.69)	0.881

**Abbreviations:** PCAT = pericoronary adipose tissue attenuation; CT-FFR = coronary computed-derived fractional flow reserve; LAD = left anterior descending; LCX = left circumflex branch; RCA = right coronary artery; HR = Hazard ratio; CI = confidence intervals; HU = Hounsfield unit; MACE = major adverse cardiovascular events.

## Data Availability

The data and supportive information are available within the article.

## LIST OF ABBREVIATIONS

PCAT: Pericoronary adipose tissue attenuation computed tomography
CTA: Computed tomography angiography
CT-FFR: Computed tomography-derived fractional flow reserve
MACE: Major adverse cardiovascular events
MI: Myocardial infraction
RCA: Right coronary artery
PCI: Percutaneous coronary intervention
CAD: Coronary artery disease

## STANDARDS OF REPORTING

STROBE guidelines were followed.
